# Influencing Factors Related to Female Sports Participation Under the Implementation of Chinese Government Interventions: An Analysis Based on the China Family Panel Studies

**DOI:** 10.3389/fpubh.2022.875373

**Published:** 2022-06-02

**Authors:** Ping Fang, Lei Sun, Shu Sheng Shi, Rizwan Ahmed Laar, Yan Lu

**Affiliations:** ^1^School of Sports Science, Nanjing Normal University, Nanjing, China; ^2^College of Physical Education, Hubei Normal University, Huangshi, China

**Keywords:** China, influencing factors, physical activity, women, government

## Abstract

**Objectives:**

Regular sports participation is a gendered phenomenon in China. Women have reported much higher constraints than men on time, partner, psychology, knowledge, and interest. This study explores personal, family, lifestyle, and health factors associated with sports participation.

**Study Design:**

This study is a cross-sectional study.

**Methods:**

Data were collected from the national reprehensive China Family Panel Studies (CFPS) database (2018) to analyze personal information, family background, lifestyle, and health in relation to women's sports participation. Multiple classification logistic regression was used to quantify the association between independent variables and sports time.

**Results:**

Women with high personal income and education, who were unmarried, in faster economic development areas have more awareness and more time for sports participation. Women who were overweight and self-rated as unattractive spent less time on sports participation. Women with a small family population and no children have more time for sports participation. Less time on the internet and moderate sleep contribute to active sports participation. Women with chronic diseases and high medical costs are less likely to participate in sports.

**Conclusions:**

Negative body aesthetic perception, the burden of family environment, modernization of lifestyle, and the normalization of sub-health are essential factors affecting women's sports participation. The government should understand the inner and outer barriers to women's participation in sports, develop policies and regulations to protect and support women's sports participation, and guide and monitor the effective implementation of women's sports activities.

## Introduction

The World Health Organization reached a consensus that health is a state of physical, mental, and social wellbeing, which indicates that the healthy development of the body is the cornerstone of everything (1). Increasing sports participation in regular physical activity to promote health is a national health priority for many developing nations (2). It is one of the effective ways to improve the health level of all the residents (3–5). Most Chinese remain inactive despite the known health benefits, and sports participation is a gendered phenomenon in China. Women have reported much higher constraints than men in terms of time, partners, psychology, knowledge, and interest (6–9).

Sedentary behavior describes the absence of sports participation. Suppose the body rapidly maladapts to insufficient physical activity and continues, resulting in substantial decreases in total and quality years of life. In that case, a sedentary lifestyle is one of the primary causes of global deaths, accounting for 5 million each year (10, 11). Therefore, reducing sedentary behavior among women and promoting sports participation may be essential strategies for reducing out-of-pocket health care expenditure in China (12).

To further develop the cause of national fitness, the Chinese government formulated a national fitness plan in two stages, first between 2011 and 2015, followed by another from 2016 to 2020, and the plan will be a “national business card.” According to the plan, by 2020, a significant increase is expected in the number of people taking part in sports, meaning people's physical quality will steadily improve. The number of people who participate in sports once a week or more can reach 700 million, and the number of people who regularly participate in sports will reach 435 million. After NFP (national fitness plan) implementation, the sports participation rate increased. Sports programs continue to enrich covering urban and rural areas. A relatively sound public service system for national fitness has improved sports participation, particularly female sports participation. In addition, the “Health China 2030” plan was implemented in 2016, aiming to build a healthy China over the next 15 years, integrating health into national policy and elevating it to the level of a national priority development strategy. Women's sports participation is an essential part of constructing a healthy China. The outline points out that strengthening health services for key populations and paying attention to the physical health of young people, women, the elderly, and other special groups. Policy encouraging women to undertake sport-related activities is more common than before. After implementing these programs, more women participated in activities such as marathons, bodybuilding, and outdoor sports.

The current study analyzes the factors that influence women's sports participation in China based on the China Family Panel Studies (CFPS) database. The study provides a guide for improving the overall level of women's health in China on a theoretical basis and shares opinions for constructing a healthy China.

## Statement of the Study

Many factors (economic, educational background, cultural impact, and domestic roles) influence women's sports participation (13, 14). The cultural background of different countries can impact women's participation in sports (15). As Laar et al. have observed, “Mass Media” and 'Religious and Cultural' factors are the most influential reasons for women's participation in sports. Other studies have highlighted a lack of knowledge, overcrowding, lack of time, long-distance, and family and financial problems as the most significant constraints women face in sports participation (16–18). Enne (19), Rosenfeld (20), and Downward et al. (21) have investigated socioeconomic, lifestyle, motivational factors, as well as the availability of sports facilities and government support related to female sports participation (20–22). Hanlon believed that a lack of professional sports knowledge significantly affects Malaysian women's sports participation (23). Sociocultural norms, family constraints, and lack of awareness about the benefits of sports strongly influenced physical activity among the different ages of US South Asian women (24).

Rodney conducted a systematic integrative literature review to identify factors affecting physical activity among African American women. These factors were classified as intrapersonal, interpersonal, and environmental (25). Studies have found that married individuals spend less time exercising and engaging in moderate to vigorous exercise than unmarried individuals. A decrease in physical activity time is more pronounced among married women than married men (26–28). In China, compared with rural areas, female participants from urban areas tended to have more leisure time for physical activity and less vigorous-intensity physical activity (29). In addition, fitness-health, enjoyment-interest, and appearance were the most critical motives, and lack of time, resources, skills, and family or friend support were the most pressing barriers to participation. Higher-income was a stronger predictor of physical activity participation in middle-aged women (16). Jing found that ill health, low energy, and lack of self-discipline affect women's physical activity (9). Choi et al. indicated that some personal and environmental factors were related to participation in physical activity. However, there is a lack of primary studies that build up organized evidence. Therefore, more studies with a prospective design should be conducted to understand the potential causes of physical activity (30). However, these studies only explore the relationship between a single variable or a few variables and female sports participation, which lacks integrity and hierarchy. The scope of research objects and the data need to be dated.

## Methods

### Data Sources

**Figure 1:**
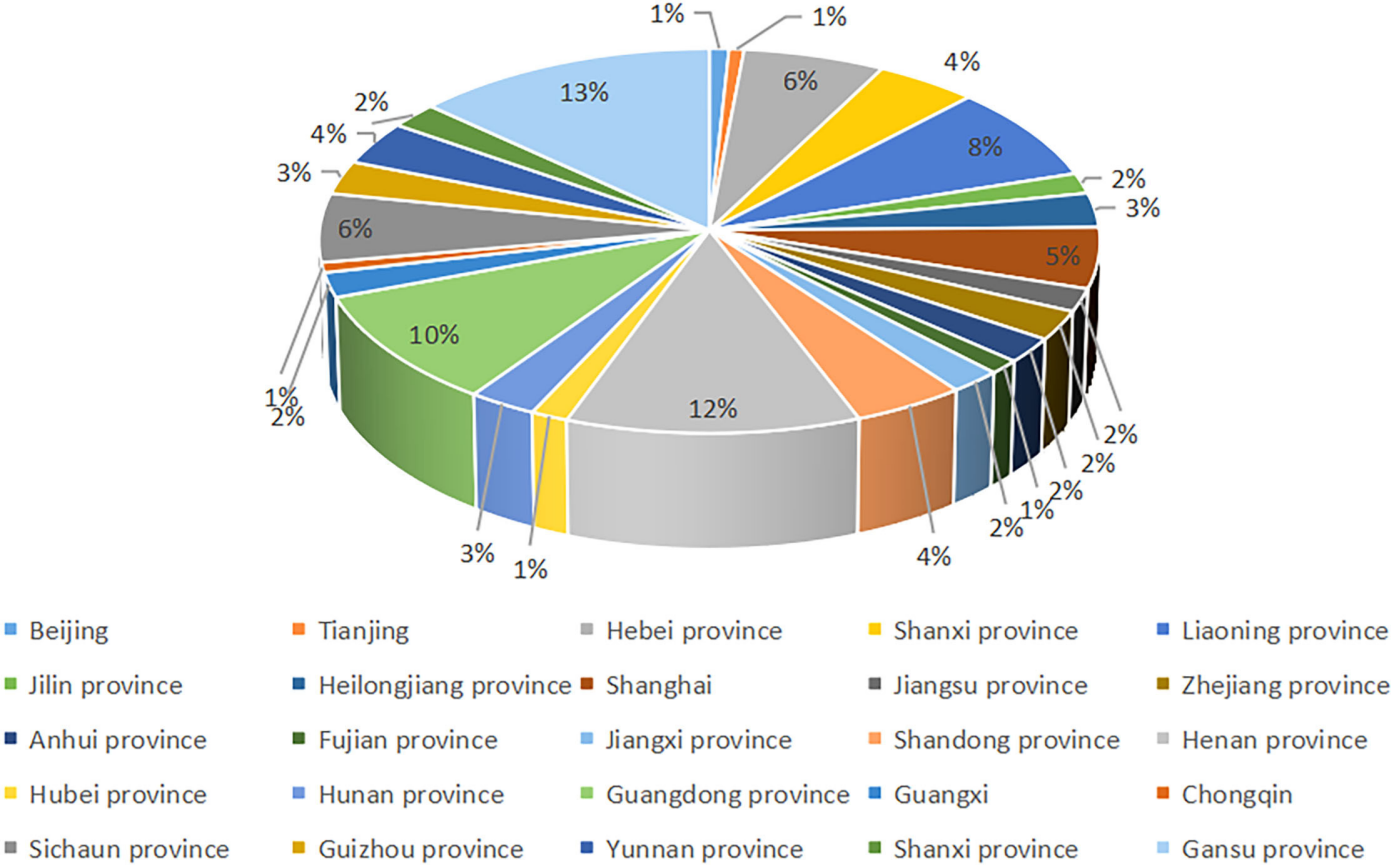
Geographical distribution of the survey participants.

We used the 2018 CFPS database, a large-scale and nationally representative survey by the Chinese Social Science Survey Center of Peking University (31). CFPS uses multi-stage sequential sampling with stratified indicators. The baseline sample covers 25 provinces/municipalities/autonomous regions (excluding Xinjiang, Tibet, Qinghai, Inner Mongolia, Ningxia, Hainan and Hong Kong, Macao, and Taiwan), representing 95% of China's population. The total sample size for 2018 was 14,241 households with 32,669 individuals. CFPS data can therefore be viewed as a nationally representative sample (Figure 1).

Women aged 16–59 were the research object (studies have proved that the elderly group over 60 years old is an active group of physical activity, so there is no specific discussion) (32). We focused on the impact of their characteristics, family environment, work background, lifestyle, and urbanization, while mainly focusing on health status during PA time. Therefore, we omitted all-male data, and 2,096 females who were not in the age group of 16–59 years, 3,338 observations with missing values were also deleted. A sample of 10,938 was obtained, and the omitting rate was 20.4%. I have employed imputation methods to compensate for missing values. Each missing value is assigned a simulated value according to the distribution of missing data, which can be seen in Supplementary Table 1.

## Specification of Variables and Model

### Variables Specification

The dependent and independent variables are in Table 1.

**Table 1:** Variable description of female participants (*N* = 10,938).

		**Variables**	**Description**	***N***	**%**
Dependent variables	Sports participation	Sports time	Never	2,202	20.13
			0–90 min	3,234	29.57
			90–180 min	3,092	28.27
			More than 180 min	2,410	22.03
	Body image	BMI	Low weight	1,329	12.2
			Overweight	2,788	25.5
			Obesity	930	8.5
			Normal	5,891	53.9
		Appearance	well	1,386	12.7
			normal	2,627	24
			not well	6,925	63.3
	Personal information	Age	16–29	2,790	25.5
			30–39	2,466	22.5
			40–49	2,755	25.2
			50–59	2,927	26.8
		Location	east	4,099	36.5
			central	3,538	32.3
			west	3,301	30.2
		Education level	Unschooled	2,989	27.3
			Elementary School	2,320	21.2
			High School	2,408	22
			Undergraduate	1,709	15.6
			Postgraduate	1,512	13.8
Independent variables		Household register	Urban	5,673	51.9
			Countryside	5,265	48.1
		Marital status	Married	6,265	52.3
			Unmarried	4,673	47.7
		Income	<1,000	7,396	67.6
			1,000–3,000	2,730	25
			>3,000	1,297	11.9
		Children	Yes	5,769	52.7
			No	5,169	47.3
	Family environment	Family population	>5	3,954	36.1
			3–5	4,182	38.2
			<3	2,792	25.5
		Frequency of caring for father	Seldom	8,951	81.8
			Sometime	1,123	10.3
			Always	864	7.9
		Frequency of caring for mother	Seldom	8,398	76.8
			Sometime	1,513	13.8
			Always	1,029	9.4
		Usage time of Internet	<1 h/day	2,704	24.7
			1–2 h/day	5,231	47.8
			2–3 h/day	1,438	13.1
			>3 h/day	1,565	14.3
	Lifestyle	Sleeping time	<6 h	4,017	36.7
			>8 h	1,371	12.5
			6–8 h	5,550	50.7
		Smoke	Yes	245	2.2
			No	10,693	97.8
		Drinking	Yes	299	2.7
			No	10,639	97.3
	Health condition	Health level	Unhealthy	2,898	26.5
			Healthy	8,040	73.5
		Chronic	Yes	2,202	20.1
			No	8,736	79.9
		Medical insurance	Yes	9,924	90.7
			No	1,014	9.3
		Medical expenses	>1,000	3,322	30.4
			500–1,000	3,434	31.4
			1–500	4,182	38.2

### Model Specification

The fitting degree test of multiple logistic regression models is based on fitting information, appropriate degree, and pseudo *R*^2^. When independent variables are not introduced in the model, the 2-fold log fit is 28227.301, reduced to 23557.214, a degree of freedom of 153, *p* < 0.01. The whole model is significant, and both the Pearson and the deviation were significantly >10%. The pseudo-deterministic coefficients for Cox and Snell, Nagelkerke, and McFadden are 0.148,0.176,0.165, respectively. These indicators show that the model fits well and fulfills the required standard (33). Therefore, it is appropriate to use the multinomial logistic regression model. The interpretation of the model reached 53.0%. A multiple logistic regression model of influencing factors of women's sports time consists of three models: model 1, model 2, and model 3. Model 1 is a comparison between level 1 and level 4. Model 2 is a comparison between level 2 and level 4, and model 3 is a comparison between level 3 and level 4. The specification of dependent and independent variables are in Supplementary Table 2.

### Statistical Analysis

Statistical analysis was performed using IBM SPSS Statistics version 24 (IBM Corp., Armonk, NY, USA), significance level set at *p* < 0.05.

## Results

### Personal Information Contributes to Independent Variables of Women's PA Time

The higher the education, the longer sports participation. Unmarried women spend more time on sports than married women. The higher the income, the more women participate in sports. Compared with women who do not participate in sports and who spend more than 180 min on sports, the age of 16–29 [1.31,95% (1.20–1.41)], 30–39 [1.58, 95% (1.53–1.63)] and 40–49 [1.22, 95% (1.53–1.63)] were 1.31, 1.58 and 1.22 times as inactive as those aged 50–59. Women in the west were 1.18 times more likely to not participate in sports than those in the East [0.85,95% (0.71–0.98)]. Urban women [1.18, 95% (1.09–1.27)] are 1.18 times more likely to not participate in sports than rural women. Obese women [1.16, 95% (1.06–1.27)] were 1.16 times more likely to not participate in sports than normal women. Unattractive women [1.52,95% (1.36–1.70)] were 1.52 times more likely to be inactive than attractive women (Table 2). The sports time was 0-90min compared with more than 180 min. The age ranges 16–29 [1.78, 95% (1.74–1.83)], 30–39 [1.67, 95% (1.61–1.73) and 40–49 [1.78, 95% (1.74–1.82)] had 0–90 min sports times of 1.78, 1.67, and 1.78 times than those aged 50–59. Urban women had a sports time of between 0 and 90 min, which was 1.49 times higher than women who lived in rural areas [0.67, 95% (0.64–0.70)]. Obese women [1.77, 95% (1.73–1.81)] had a sports time of between 0 and 90 min, which was 1.77 times more than normal (Table 2).

**Table 2:** Multiple Logistic Regression models of contributing factors of female participation in physical activity (personal information).

**Independent variable**		**Model 1**		**Model 2**		**Model 3**	
		**RC (SEM)**	**RRR (95%CI)**	**RC (SEM)**	**RRR (95%CI)**	**RC (SEM)**	**RRR (95%CI)**
Age	16–29 years old	1.15** (0.14)	1.31 (1.20–1.42)	0.89** (0.12)	1.78 (1.74–1.83)	0.71 (0.18)	1.73 (1.53–1.97)
	30–39 years old	0.75** (0.12)	1.58 (1.53–1.63)	1.25** (0.10)	1.67 (1.61–1.73)	0.72 (0.16)	0.68 (0.59–0.77)
	40–49 years old	0.26** (0.09)	1.22 (1.12–1.33)	0.31** (0.08)	1.78 (1.74–1.82)	0.41 (0.12)	1.11 (0.96–1.28)
	50–59 years old	Referent	–	Referent	–	Referent	–
Location	East	−0.34** (0.07)	0.85 (0.72–0.98)	0.12 (0.07)	1.14 (1.09–1.19)	−0.16 (0.10)	1.65 (1.46–1.87)
	Central	−0.24** (0.07)	0.98 (0.91–1.05)	0.15 (0.07)	1.34 (1.29–1.39)	−0.03 (0.10)	0.65 (0.57–0.73)
	West	Referent	–	Referent	–	Referent	–
Education level	Unschooled	0.67** (0.19)	1.70 (1.58–1.84)	0.31** (0.08)	1.70 (1.61–1.79)	0.43** (0.12)	1.52 (1.46–1.58)
	Elementary School	0.56** (0.18)	1.53 (1.48–1.58)	0.40** (0.13)	1.66 (1.60–1.72)	0.38** (0.12)	1.47 (1.14–1,89)
	High School	0.27** (0.12)	1.09 (0.97–1.23)	0.21** (0.14)	1.23 (1.04–1.46)	0.25** (0.17)	1.82 (1.56–2.12)
	Undergraduate	0.28** (0.20)	1.07 (0.91–1.25)	0.005 (0.15)	0.84 (0.76–0.92)	−0.08 (0.19)	1.62 (1.32–1.97)
	Postgraduate	Referent	–	Referent	–	Referent	–
Household register	Urban	0.17* (0.07)	1.18 (1.09–1.27)	−0.60** (0.06)	0.67 (0.64–0.70)	−0.23** (0.11)	0.79 (0.65–0.97)
	Rural	Referent	–	Referent	–	Referent	–
Marital status	Married	1.32** (0.15)	2.09 (1.57–2.61)	0.98** (0.16)	2.66 (1.94–3.64)	1.15** (0.12)	2.02 (1.76–2.33)
	Unmarried	Referent	–	Referent	–	Referent	–
Income	<1,000	0.59** (0.12)	1.55 (1.43–1.70)	0.57** (0.11)	1.66 (1.54–1.81)	1.59** (1.12)	1.55 (1.42–1.72)
	Between 1,000 and 3,000	0.23** (0.14)	1.81 (1.64–2.04)	0.21** (0.11)	1.86 (1.81–0.91)	0.09 (0.14)	1.41 (1.22–1.62)
	More than 3,000	Referent	–	Referent	–	Referent	–
BMI	Low weight	0.04 (0.14)	1.32 (1.17–1.49)	0.04 (0.10)	0.84 (0.78–0.91)	0.04 (0.14)	1.16 (0.94–1.42)
	Overweight	0.09** (0.08)	1.10 (0.94–1.27)	−0.13 (0.09)	1.31 (1.26–1.37)	−0.05 (0.12)	0.86 (0.76–0.98)
	Obesity	0.59** (0.13)	1.16 (1.06–1.27)	0.81** (0.11)	1.77 (1.73–1.81)	0.25* (0.13)	1.12 (0.97–1.30)
	Normal	Referent	–	Referent	–	Referent	–
Appearance	Not well	1.17** (0.09)	1.52 (1.36–1.70)	1.60** (0.07)	1.19 (1.13–1.27)	0.17 (0.12)	0.41 (0.29–0.57)
	Normal	0.18 (0.06)	1.08 (0.98–1.20)	0.23 (0.04)	0.66 (0.62–0.70)	0.08 (0.05)	1.59 (1.39–1.82)
	well	Referent	–	Referent	–	Referent	–

*RC, regression coefficient; RRR, Relative Risk ratio; BMI, body mass index*,

* *p < 0.05,*

** *p < 0 .01 for the referent*.

### Family Background Contributes to Independent Variables of Women's PA Time

Women without children were 1.136 times more likely to not participate in sports than women with children [0.88, 95% (0.81–0.95)], but women with children [1.49,95% (1.42–1.56)] were 1.49 times more likely to exercise for 0–90 min than women with children (Table 3). Women with families of more than five were more likely to be inactive than women with families of less than three. Women who never took care of their fathers [1.50, 95% (1.39–1.61)] had 0–90 min sports time and were 1.5 times more likely to undertake exercise than women who took care of their fathers all the time. Women who cared for their mothers were more likely to participate in sports than women who never cared for their mothers [0.80, 95% (0.73–0.87)] (Table 3).

**Table 3:** Multiple Logistic Regression models of contributing factors of female participation in physical activity (family background).

**Independent variable**		**Model 1**		**Model 2**		**Model 3**	
		**RC (SEM)**	**RRR (95%CI)**	**RC (SEM)**	**RRR (95%CI)**	**RC (SEM)**	**RRR (OR95%CI)**
Children	Yes	0.19** (0.08)	0.88 (0.81–0.95)	0.21** (0.07)	1.49 (1.42–1.56)	−0.04 (0.10)	0.67 (0.59–0.75)
	No	Referent	–	Referent	–	Referent	–
Family population	More than 5	0.62** (0.10)	1.95 (1.87–2.04)	0.17* (0.07)	1.44 (1.39–1.50)	0.27 (0.13)	1.30 (1.14–1.47)
	Between 3 and 5	0.32** (0.08)	1.65 (1.61–1.70)	0.12 (0.09)	0.75 (0.71–0.79)	0.12 (0.11)	0.81 (0.72–0.91)
	Less than 3	Referent	–	Referent	–	Referent	–
Frequency of caring for father	Seldom	0.16 (0.15)	0.72 (0.65–0.79)	0.33* (0.13)	1.50 (1.39–1.61)	0.05 (0.18)	0.80 (0.68–0.94)
	Sometime	−0.23 (0.18)	1.42 (1.26–1.60)	−0.23 (0.16)	0.65 (0.59–0.72)	−0.45 (0.23)	1.08 (0.86–1.34)
	Always	Referent	–	Referent	–	Referent	–
Frequency of caring for mother	Seldom	0.34** (0.13)	0.80 (0.73–0.87)	0.14* (0.11)	1.40 (1.32–1.49)	0.10 (0.16)	0.76 (0.66–0.87)
	Sometime	0.30 (0.16)	1.34 (1.20–1.48)	−0.37 (0.14)	0.69 (0.64–0.75)	0.10 (0.19)	1.23 (1.04–1.47)
	Always	Referent	–	Referent	–	Referent	–

*RC, Regression coefficient; RRR, Relative Risk ratio*,

* *p < 0.05*,

** *p < 0.01 for the referent*.

### Lifestyle and Health Conditions Contributing Independent Variables of Women's PA Time

For sports participation times between 90 and 180 min, women who used the internet for <1 h [1.39, 95% (1.21–1.60)], 1–2 h [1.45, 95% (1.36–1.55)], and 2–3 h [1.63, 95% (1.56–1.71)] were 1.39, 1.45, and 1.64 times more likely to exercise than women who used the internet for 3 h a day. Women who slept less than 6 hours [1.39, 95% (1.24–1.64)] and more than 8 hours [1.74,95% ([1.53–1.95)] and were 1.39 and 1.74 times more likely to exercise than women who slept between 6–8 h. Women who were unhealthy, with chronic diseases, and who paid high medical expenses spent less time playing sports than healthy women (Table 4).

**Table 4:** Multiple Logistic Regression models of contributing factors of female participation in physical activity (lifestyle and health condition).

**Independent variable**		**Model 1**		**Model 2**		**Model 3**	
		**RC (SEM)**	**RRR (95%CI)**	**RC (SEM)**	**RRR (95%CI)**	**RC (SEM)**	**RRR (95%CI)**
Usage time of internet	<1 h a day	−0.26 (0.14)	1.50 (1.37–1.63)	0.07 (0.12)	0.70 (0.65–0.74)	−0.49** (0.17)	1.39 (1.21–1.60)
	Between 1 and 2 h a day	−0.08 (0.13)	0.62 (0.58–0.67)	0.15 (0.11)	0.98 (0.91–0.15)	−0.39* (0.16)	1.45 (1.36–1.55)
	Between 2 and 3 h a day	−0.25 (0.16)	1.27 (1.11–1.44)	−0.11 (0.14)	0.69 (0.63–0.76)	−0.53** (0.18)	1.63 (1.56–1.71)
	More than 3 h a day	Referent	–	Referent	–	Referent	–
Sleeping time	<6 h	0.33** (0.078)	1.39 (1.24–1.64)	−0.02 (0.10)	0.75 (0.72–0.79)	−0.09 (0.14)	1.00 (0.88–1.14)
	More than 8 h	0.55** (0.11)	1.74 (1.53–1.95)	0.22* (0.09)	1.39 (1.33–1.45)	−0.09 (0.13)	1.00 (0.88–1.13)
	Between 6 and 8 h	Referent	–	Referent	–	Referent	–
Smoke	Yes	−0.43 (0.26)	0.85 (0.62–1.18)	0.06 (0.18)	0.99 (0.85–1.14)	−0.64 (0.35)	0.60 (0.33–1.10)
	No	Referent	–	Referent	–	Referent	–
Drinking	Yes	−0.12 (0.22)	1.33 (0.92–1.92)	−0.32 (0.17)	0.92 (0.80–1.05)	−0.18 (0.26)	0.93 (0.61–1.42)
	No	Referent	–	Referent	–	Referent	–
Health level	Unhealthy	0.39** (0.08)	1.58 (1.37–1.79)	0.23 (0.07)	1.15 (1.09–1.21)	0.10 (0.11)	1.28 (1.09–1.49)
	Healthy	Referent	–	Referent	–	Referent	–
Chronic diseases	Yes	2.38** (0.11)	4.65 (4.35–4.96)	0.49** (0.11)	0.47 (0.43–0.51)	0.78** (0.15)	0.66 (0.54–0.80)
	No	Referent	–	Referent	–	Referent	–
Medical insurance	Yes	0.10 (0.13)	1.16 (1.00–1.34)	0.03 (0.10)	1.02 (0.95–1.09)	0.02 (0.14)	0.83 (0.68–1.01)
	No	Referent	–	Referent	–	Referent	–
Medical expenses	More than 1,000	0.42** (0.09)	0.96 (0.88–1.04)	0.07 (0.07)	0.92 (0.88–0.96)	−0.08 (0.10)	1.15 (1.01–1.31)
	Between 500 and 1,000	0.37** (0.09)	0.81 (0.75–0.87)	0.41** (0.07)	1.32 (1.27–1.37)	0.09 (0.10)	0.78 (0.69–0.89)
	Between 1 and 500	Referent	–	Referent	–	Referent	–

*RC, Regression coefficient; RRR, Relative Risk ratio*,

* *p < 0.05*,

** *p < 0.01 for the referent*.

## Discussion

This study explored the associations of women's sports participation with personal-related, family environment-related, and lifestyle-related factors in 10,938 Chinese women aged between 16 and 59 years old. We found that women aged 50–59 participate in sports for longer because they are less competitive at work and have more leisure time toward retirement. Other age groups are busy with school, work and family. This finding was similar to those in research by Santos (34). Due to urbanization factors, western China's economic and cultural development lags behind central China's, and there is insufficient awareness of female sports participation in the west. Meanwhile, higher education gives individuals more spare time for exercise. As some studies have shown, the higher the education level, the more positive the attitude toward exercise, and the more regular the sports participation (35, 36). Studies have found that married women spend less time on sports than unmarried women. Because married women are family-oriented and devote less time to leisure (37), especially in married women than men (26–28). However, married women are more likely to spend 0–90 min exercising with their children and other family members. A higher income level may serve as a marker for more discretionary time or resources that enable Chinese women to engage in higher levels of sports. This finding is consistent with previous studies among women in general (14).

A survey in Chinese provinces found that 34% of adults between the ages of 20 and 69 are overweight, 18.9% are overweight, and 2.9% of people in China are obese (38). Significantly, physical inactivity was associated with being overweight/obesity (39). Body mass index and waist circumference have increased considerably in women over 20 years. In 2018, overweight and obese women had less time for sports participation. Sports are often considered an effective way to reduce fat and improve body shape. Even though overweight and obese people should participate, overweight and obese women are sometimes unwilling to participate in sports (40, 41). They tend to associate this experience with low self-esteem, embarrassment, and feelings of vulnerability (42, 43), meaning individuals are likely to give up or avoid participation in sports due to psychological stress and anxiety. A systematic integrative literature review to identify factors to sports participation classified the elements as intrapersonal, interpersonal, and environmental (25). Women with poor physical appearance have anxiety when participating in sports, which can take the form of concern or fear related to being negatively evaluated by others in situations where the physical appearance of an individual can be assessed by others.

We found that the family population is also an essential aspect of the family environment. Our study indicates families of less than three persons are more likely to participate in sports. The women need to take care of all family member's needs, and women with small families spend their time undertaking low-cost leisure activities such as sports in China. These findings are consistent with those of a previous study (44). Women who regularly care for their mothers have time for participation in sports since women who care for their mothers also insisted on accompanying them to participate in appropriate sports activities. This is the same need for women in many developing countries to have family responsibilities and care ethics (45).

Thirdly, we found that moderate sleep helps with active participation in sports. Being addicted to the internet, playing games, watching TV, and other bad life habits significantly reduce women's sports participation time. There was a negative association between screen time and sports participation (46, 47). Moderate sleep is more helpful for women to be active in sports participation than low and high sleeping time (48). This is consistent with existing studies, which indicate that inadequate sleep and excessive sleep have adverse effects on women's sports participation time (49).

**Figure 2:**
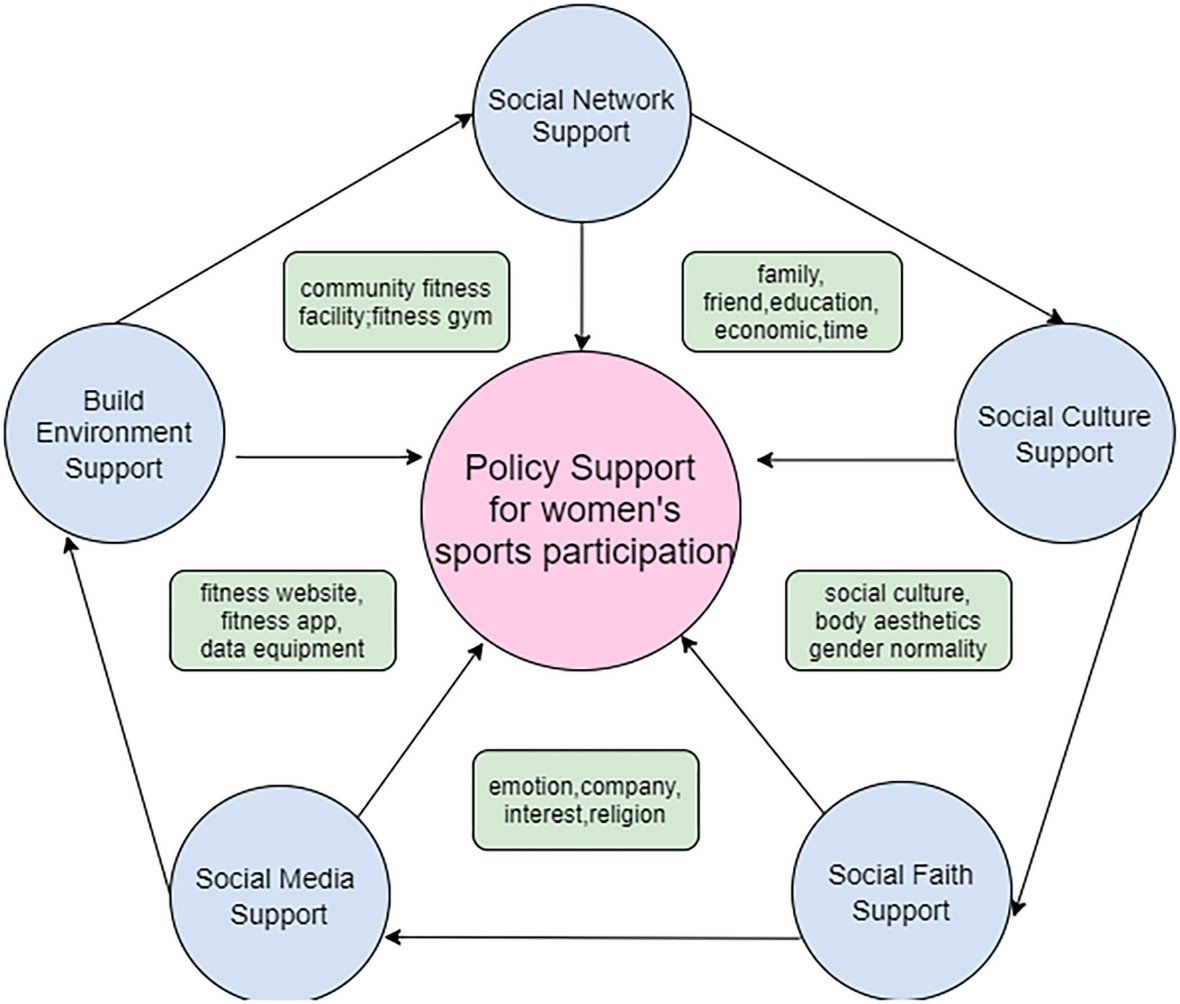
Policy recommendations to promote Chinese female sports participants.

Fourth, we also found that physical inactivity increases the health risks and disease burden in China (50). Health level, chronic diseases, and medical expenditure have significant effects on women's participation in sports. Unhealthy women have a higher probability of not participating in sports than healthy women. Individuals with limited exercise capacity due to the disease were less likely to participate in sports, especially for long periods. This study does not support the significant role of health insurance in sports participation, probably because China's current health insurance system is relatively complete, with broad coverage and many types, and the effect of having or not having health insurance on sports participation is not significant. This study indicates that women with chronic diseases have less will to participate in sports, and increasing sports participation could reduce total medical costs to some extent (51). Sports participation leads to metabolic disorders, obesity, type 2 diabetes, and other diseases. Studying the correlates of sports time is an essential prerequisite for designing relevant policies and effective programs. The current study can give a fresh understanding of Chinese women's physical activity participation rate and barriers. It will help promote the health of women and the next generation and ultimately contribute to realizing the “Healthy China” strategy. The government should understand the inner and outer barriers to women's participation in sports, develop policies and regulations to protect and support women's sports participation, and guide and monitor the implementation and effective implementation of women's sports activities (Figure 2).

There were some limitations to this study. The first is that no information was collected on the type of sports activities undertaken by women. Secondly, some data were self-reported, making them prone to reporting bias, which may add some measurement error. Another important factor is the lack of discussion on the impact of women's work environment (working hours, type of work, length of work) on sports participation time.

## Conclusion

Women with high personal income and education, who are unmarried, in faster economic development areas have more vital awareness and more time for sports participation. Women who are overweight and unattractive have anxiety about sports participation. Women with a small family population and no children have more time for sports participation. Spending less time on the internet and having moderate sleep contribute to active sports participation. Chronic diseases and high medical expenses give women no time for sports participation. Overall, negative body aesthetic perception, the duality of family and social work, the modernization of lifestyle, and the normalization of sub-health are essential factors affecting women's sports participation.

The government should develop policies and regulations to protect and support women's sports participation in the following areas, and guide and monitor the effective implementation of women's sports activities. Firstly, developing social network support policies to increase solid relational support, such as family, friends, emotional support, time, and economic support. Secondly, developing social environment support policies to alleviate social gender norms and socio-cultural restrictions in sports, could help women establish better body aesthetic concepts, and enhance body confidence through physical exercise. Improving the safety of fitness communities, transportation accessibility convenience, and the comprehensiveness of sports facilities could also increase women's willingness to participate in sports. Finally, improving social media support to increase the positive impact of social media programs through fitness websites, fitness APPs, and data monitoring software could also help enhance women's participation in sports.

## Data Availability Statement

The datasets presented in this study can be found in online repositories. The names of the repository/repositories and accession number(s) can be found in the article/Supplementary Material.

## Author Contributions

PF had the idea for the article, drafted the article, wrote the sections of the article and analyzed the data, and conducted and reported the work described in the article. LS participated in the discussions, organized the planning, and continued to write and revised the article. YL checked supplementary materials. PF and SS are responsible for the overall content as guarantors. All authors read and approved the final manuscript.

## Funding

This research was supported by the National Social Science Fund of China 2021 (21BTY024), a Major Project of Philosophy and Social Science Research in Colleges and Universities of Jiangsu Province 2020 (2020SJZDA128), and a Social Science Fund Project of Jiangsu Province 2020 (20TYB001).

## Conflict of Interest

The authors declare that the research was conducted in the absence of any commercial or financial relationships that could be construed as a potential conflict of interest.

## Publisher's Note

All claims expressed in this article are solely those of the authors and do not necessarily represent those of their affiliated organizations, or those of the publisher, the editors and the reviewers. Any product that may be evaluated in this article, or claim that may be made by its manufacturer, is not guaranteed or endorsed by the publisher.
