# Supplementary material for: Influencing Factors Related to Female Sports Participation Under the Implementation of Chinese Government Interventions: An Analysis Based on the China Family Panel Studies
Source: Front Public Health. 2022 Jun 2;10:875373. doi: 10.3389/fpubh.2022.875373 (PMC9201213; doi:10.3389/fpubh.2022.875373)
Supplement: Supplementary file 1 [file Data_Sheet_1.pdf]

Table S1 Outcome variable

	Outcome variable	The number of missing values to replace	Case numbers for non-missing values		Number of active cases	Create a function
			First	Last		
1	Sports time	1602	1	10938	10938	SMEAN (Sports time)
2	BMI	2318	1	10938	10938	SMEAN (BMI)
3	Appearance	3303	1	10938	10938	SMEAN (Appearance
4	Age	2188	1	10938	10938	SMEAN (Age)
5	Location	2120	1	10938	10938	SMEAN (Location)
6	Education level	2305	1	10938	10938	SMEAN (Education level)
7	Household register	2417	1	10938	10938	SMEAN (Household register)
8	Marital status	1550	1	10938	10938	SMEAN (Marital status)
9	Income	2077	1	10938	10938	SMEAN (Income)
10	Children	2582	1	10938	10938	SMEAN (Children)
11	Family population	2776	1	10938	10938	SMEAN (Family population)
12	Frequency of caring for father	3622	1	10938	10938	SMEAN (Frequency of caring for father)
13	Frequency of caring for mother	2780	1	10938	10938	SMEAN (Frequency of caring for mother)
14	Usage time of Internet	3352	1	10938	10938	SMEAN (Usage time of Internet)
15	Sleeping time	2282	1	10938	10938	SMEAN (Sleeping time)
16	Smoke	2544	1	10938	10938	SMEAN (Smoke)
17	Drinking	1423	1	10938	10938	SMEAN (Drinking)
18	Health level	1354	1	10938	10938	SMEAN (Health level)
19	Chronic	1135	1	10938	10938	SMEAN (Chronic)
20	Medical insurance	1104	1	10938	10938	SMEAN (Medical insurance)
21	Medical expenses	1156	1	10938	10938	SMEAN (Medical expenses)
