# Supplementary material for: Influencing Factors Related to Female Sports Participation Under the Implementation of Chinese Government Interventions: An Analysis Based on the China Family Panel Studies
Source: Front Public Health. 2022 Jun 2;10:875373. doi: 10.3389/fpubh.2022.875373 (PMC9201213; doi:10.3389/fpubh.2022.875373)
Supplement: Supplementary file 2 [file Data_Sheet_2.pdf]

S2 The specification of dependent and independent variables

The dependent variable

Sports participation time: In CFPS, women were directly asked for how many hours of physical activity they engaged in per week.

Independent variables

BMI: CFPS directly investigated the height and weight of the respondents and calculated the ratio of weight to height squared. According to Asian standards, the respondents were divided into four groups according to BMI: low weight (BMI < 18.5), normal (18.5 ≤ BMI ≤ 23.9), overweight (24 ≤ BMI ≤ 27.9) and obese (BMI ≥ 28).

Appearance: This study adopts the self-rated appearance of the respondents in CFPS, which has a seven-level standard ranging from "very poor" to "very well". According to the needs of this study, the appearance is divided into three levels: "not well", "normal" and "well".

Age: In this study, CFPS age grouping method was used to divide adult females aged 16-59 into four groups, namely, 16-29 years old, 30-39 years old, 40-49 years old, and 50-59 years old.

Location: The CEPS are divided into eastern (11), central (9), and western (5) provinces according to their geographical location.

Education level: This study divides them into five levels: unschooled, elementary school, high school, undergraduate and postgraduate.

Household register: The CFPS has "What is your current household registration place?" to investigate the household registration status of women. It is mainly divided into urban and countryside.

Marital status: Used the CFPS "Your current marital status" to measure women's marital status, which was divided into married and unmarried.

Income: CFPS has "your monthly after-tax income (yuan/month)" to measure women personal income status. Based on the three-point method, we divided female work income into three categorical variables.

Children: Children were determined according to the number of children in the CFPS parents' proxy questionnaire.

Family population: We used the "how many people are in your family" to measure the family population.

Frequency of caring for father: The study used the "How often do you take care of your father's household, food and daily activities?" to measure how often care for her father.

Frequency of caring for mother: The study used the "How often do you take care of your mother's household, food and daily activities?" to measure how often care for her mother.

Usage time of Internet: Used "How many hours per week do you spend online in your spare time?" to measure the average amount of time women spend on the Internet outside of work each day

Sleeping time: Used "About how many hours do you sleep each day?" to measure how long the women slept each day.

Smoke: The CFPS "Are you smokers" was used to determine whether women smoked.

Drinking: Using the CFPS "Have you had more than 3 drinks per week in the past month?" Determine a woman is drinking excessively or not.

Health level: The CFPS questioned "How do you consider your health?" to evaluate Health level (self-rated).

Chronic: This study used the CFPS "Have you had a chronic disease diagnosed by a doctor in the past six months?" to measure chronic disease in women

Medical insurance: We used the CFPS "Do you have health insurance" to judge women's health insurance.

Medical expenses: We used "How much additional money have you spent in the last 12 months due to injuries?" to measure women's health care costs.
